# Epigenetic Dysregulation in Mesenchymal Stem Cell Aging and Spontaneous Differentiation

**DOI:** 10.1371/journal.pone.0020526

**Published:** 2011-06-09

**Authors:** Zhilong Li, Chenxiong Liu, Zhenhua Xie, Pengyue Song, Robert C. H. Zhao, Ling Guo, Zhigang Liu, Yaojiong Wu

**Affiliations:** 1 Life Science Division, Graduate School at Shenzhen, Tsinghua University, Shenzhen, China; 2 State Key Laboratory of Respiratory Disease for Allergy at Shengzhen University, School of Medicine, Shenzhen University, Shenzhen, China; 3 Center of Excellence in Tissue Engineering, Department of Cell Biology, Institute of Basic Medical Sciences, Chinese Academy of Medical Sciences and School of Basic Medicine, Peking Union Medical College, Beijing, China; Instituto Nacional de Câncer, Brazil

## Abstract

**Background:**

Mesenchymal stem cells (MSCs) hold great promise for the treatment of difficult diseases. As MSCs represent a rare cell population, *ex vivo* expansion of MSCs is indispensable to obtain sufficient amounts of cells for therapies and tissue engineering. However, spontaneous differentiation and aging of MSCs occur during expansion and the molecular mechanisms involved have been poorly understood.

**Methodology/Principal Findings:**

Human MSCs in early and late passages were examined for their expression of genes involved in osteogenesis to determine their spontaneous differentiation towards osteoblasts *in vitro*, and of genes involved in self-renewal and proliferation for multipotent differentiation potential. In parallel, promoter DNA methylation and hostone H3 acetylation levels were determined. We found that MSCs underwent aging and spontaneous osteogenic differentiation upon regular culture expansion, with progressive downregulation of TERT and upregulation of osteogenic genes such as Runx2 and ALP. Meanwhile, the expression of genes associated with stem cell self-renewal such as Oct4 and Sox2 declined markedly. Notably, the altered expression of these genes were closely associated with epigenetic dysregulation of histone H3 acetylation in K9 and K14, but not with methylation of CpG islands in the promoter regions of most of these genes. bFGF promoted MSC proliferation and suppressed its spontaneous osteogenic differentiation, with corresponding changes in histone H3 acetylation in TERT, Oct4, Sox2, Runx2 and ALP genes.

**Conclusions/Significance:**

Our results indicate that histone H3 acetylation, which can be modulated by extrinsic signals, plays a key role in regulating MSC aging and differentiation.

## Introduction

Mesenchymal stem cells (MSCs) are self-renewing and expandable stem cells [1], [2], [3]. In order to compare and contrast study outcomes from different research groups, the Mesenchymal and Tissue Stem Cell Committee of the International Society for Cellular Therapy proposed a minimal criterion to define human MSCs. First, MSCs must be plastic-adherent when maintained in standard culture conditions. Second, MSCs must be lineage-negative and express CD105, CD73 and CD90. Third, MSCs must differentiate to at least osteoblasts, adipocytes and chondroblasts *ex vivo* [3].

Increasing evidence has suggested profound therapeutic potential of MSCs for a variety of diseases such as myocardial infarction [4], [5], [6], [7], neural diseases [8], [9] strokes [10], and wound healing [11], [12]. Moreover, allogeneic MSCs have shown low immunogenicity and immunosuppressive properties [13], [14], [15]. Due to encouraging preclinical results, numerous clinical trials for a variety of diseases are underway [16], [17], [18], [19], [20].

MSCs represent as a rare cell population in the bone marrow (BM) and other tissues. BM is the major source of MSCs, where they represent only approximately 0.001% to 0.01% of the nucleated cells, about 10-fold less abundant than hematopoietic stem cells (HSCs). Therefore, *ex vivo* expansion of MSCs is an indispensable procedure to obtain sufficient amounts of cells for MSC-based therapies and tissue engineering. MSCs are capable of proliferating in culture [1], [2], and they are genetically stable when undergoing limited *ex vivo* expansion [21]. However, recent studies suggest MSCs age rapidly in culture and undergo considerable property changes. This has raised concerns over the effect and safety of MSC-based therapies [22], [23]. More importantly, the molecular mechanisms underlying phonotypical changes of MSCs during culture expansion are unclear.

In this study, we found that MSCs underwent considerable epigenetic and gene expressional alterations during culture expansion, even though the morphological changes are modest. The expression of osteogenic genes increased progressively with successive passages of MSCs, while the expression of “stemness” genes such as Oct4 and Sox2 declined markedly. In accordance with these changes were epigenetic dysregulations, with histone H3 acetylation in particular. Basic fibroblast growth factor (bFGF) modulated histone H3 acetylation in telomerase reverse transcriptase (TERT), Oct4, Sox2, Runx2 and alkaline phosphatase (ALP) genes, promoted MSC proliferation and suppressed its spontaneous osteogenic differentiation. *Ex vivo* culture of MSCs also caused changes in methylation levels in CpG islands in the promoter and exon 1 regions in most of these genes, but the changes did not coincide with expressional changes of the corresponding genes. Therefore, our results suggest that acetylation of histone H3 modulates the expression of critical genes in MSCs, thereby regulating their behavior.

## Results

### Characterization of MSCs

**Figure 1:**
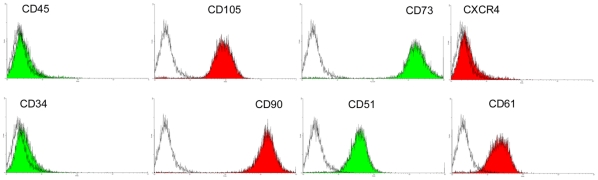
Fluorescence-activated cell sorting (FACS) analysis of MSCs. Passage 3 MSCs were analyzed by FACS after staining with FITC- or PE-conjugated control isotype IgG (gray peaks) or antibodies against indicated cell surface proteins (filled red or green peaks).

**Figure 2:**
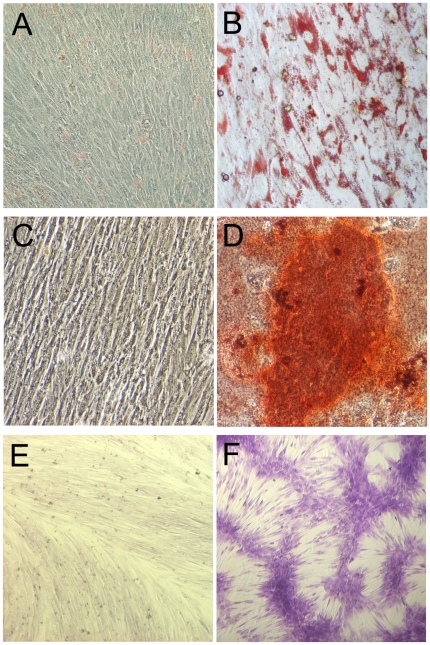
Differentiation of MSCs. Cultured in appropriate induction media, (A & B) MSCs differentiated into adipocytes (after oil red staining, A represents non-induced and B represents induced), (C & D) osteoblasts (after Alizarin Red S staining, C represents non-induced and D represents induced), and (E & F) chondrocytes (after Alcian Blue staining, E represents non-induced and F represents induced).

Fluorescence activated cell sorting (FACS) analysis of our MSCs showed that they were negative for lineage cell markers such as CD34 and CD45, and were strongly positive for CD105, CD73 and CD90, exhibiting typical immunophenotypic features of MSCs (Fig. 1). The cells also expressed CD51 and CD61 (Fig. 1). After induction in appropriate media, MSCs differentiated into adipocytes (Fig. 2B), osteoblasts (Fig. 2D) and chondrocytes (Fig. 2F).

### Spontaneous differentiation and downregulation of pluripotent genes in MSCs during culture expansion

**Figure 3:**
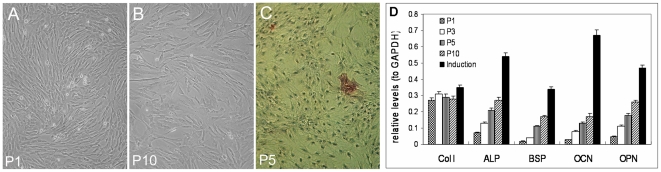
Morphological changes and spontaneous estrogenic differentiation of MSCs. Cultured in growth medium, (A & B) MSCs became larger and fatter upon expansion, (C) A few cells became extremely larger and flatter and were positive (in red) for alkaline phosphatase (ALP) stain. (D) Real-Time PCR analysis showed that the expression of genes associated with osteogenesis such as collagen type I (Col I), alkaline phosphatase (ALP), bone sialoprotein (BSP), osteocalcin (OCN) and osteopontin (OPN) increased progressively with cell passages. P1 to P10 represents MSC passage number. Induction indicates MSCs after incubation with osteogenic induction medium for 14 days.

**Figure 4:**
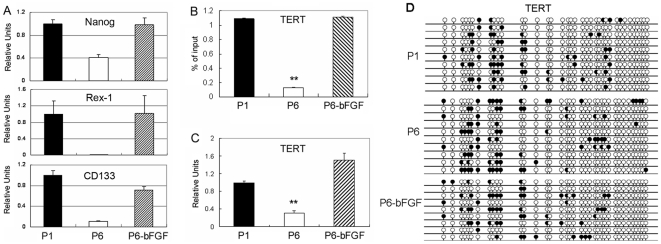
Gene expression, histone acetylation and DNA methylation in early and late passage MSCs. (A) Real-Time PCR analysis of the expression of Nanog, REX1 and CD133 in culture passage (P) 1, passage 6 MSCs cultured in growth medium or passage 6 MSCs growth medium supplemented with bFGF (P6-bFGF). bFGF treatment started from passage 1 cells. (B) TERT histone H3 acetylation (** *P*<0.01 versus P1 and P6 in bFGF-supplemented culture). (C) TERT gene expression (Real-Time PCR analysis) and, (D) DNA methylation in CpG islands in the promoter region of TERT in passage 1 and 6 MSCs cultured in growth medium versus passage 6 MSCs cultured in growth medium supplemented with bFGF (P6-bFGF).

**Figure 5 pone-0020526-g005:**
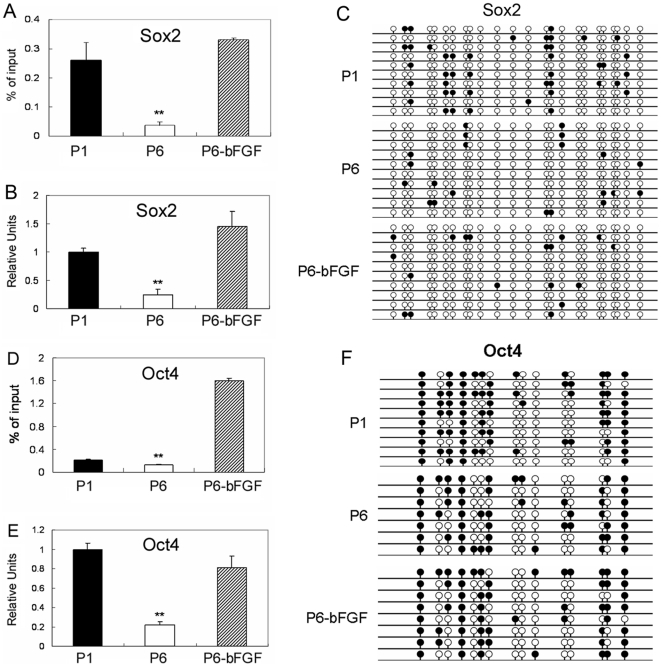
Multipotent gene expression, histone acetylation and DNA methylation in early and late passage MSCs. Gene expression, histone acetylation and DNA methylation. (A) Sox2 and (D) Oct4 histone H3 acetylation (** *P*<0.01); (B) Sox2 and (E) Oct4 gene expression (Real-Time PCR analysis), and (C) DNA methylation in CpG islands in the promoter region and exon 1 region of Sox2 and (F) Oct in passage (P) 1 and 6 MSCs cultured in growth medium versus passage 6 MSCs cultured in growth medium supplemented with bFGF (P6-bFGF).

Cultured in growth medium, MSCs underwent modest but progressive morphological changes with successive passages. The cells became larger and fatter in general (Fig. 3A and B). Meanwhile, a small portion of cells became extremely larger and flatter, which were similar to osteoblasts in morphology and positive for ALP stain (Fig. 3C), indicating the presence of spontaneous differentiation of MSCs into osteoblasts. In accordance with changes in morphology, the expression of genes associated with osteogenesis such as collagen type I, ALP, bone sialoprotein (BSP), osteocalcin (OCN) and osteopontin (OPN) increased progressively with successive cell passages (Fig. 3D), while the expression of genes associated with stem cell pluripotency and proliferation decreased markedly, which included Oct4, Sox2, Nanog, REX1, CD133 and TERT (Fig. 4A and C; Fig. 5B and E).

### Epigenetic dysregulation of MSCs

**Figure 6:**
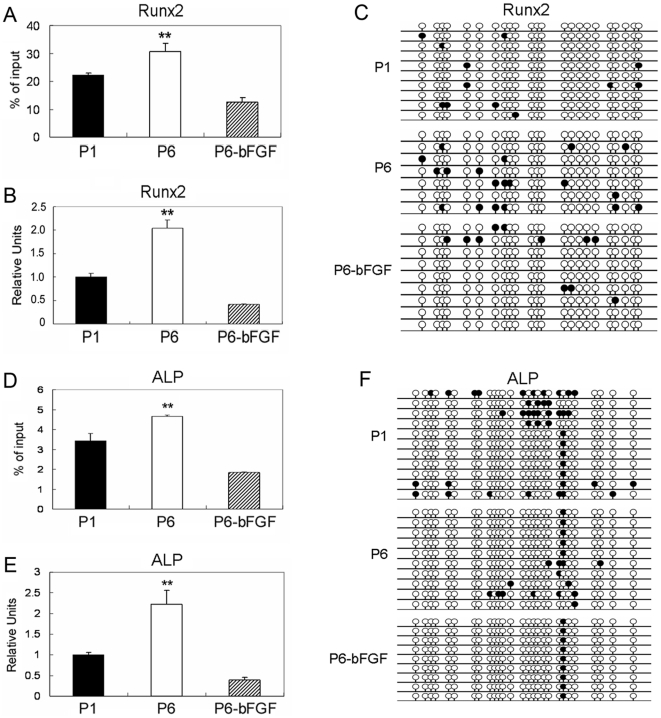
Osteogenic gene expression, histone acetylation and DNA methylation in early and late passage MSCs. (A) Runx2 and (D) ALP histone H3 acetylation (** *P*<0.01), (B) Runx2 and (E) ALP gene expression (Real-Time PCR analysis), and (C) DNA methylation in CpG islands in the promoter region of Runx2 and (F) in the in the promoter region and exon 1 region of ALP in passage (P) 1 and 6 MSCs cultured in growth medium versus passage 6 MSCs cultured in growth medium supplemented with bFGF (P6-bFGF).

Previous studies have shown that MSCs are genetically stable after limited expansion [21]. So we investigated whether MSCs underwent epigenetic changes during culture expansion, with an emphasis on promoter DNA methylation and histone H3 acetylation. We found dramatic alterations in histone H3 acetylation in K 9 and K14 in TERT, Sox2, Oct4, Runx2 and ALP genes in replicating MSCs when comparing passage 1 with passage 6 MSCs (Fig. 4B; Fig. 5A, 5D, 6A and 6D). Downregulation in the expression of TERT, Sox2 and Oct4 genes were closely associated with decreases in H3 acetylation levels in the promoter regions of the corresponding genes (Fig. 4B and 4C; Fig. 5A, B, D and E). Meanwhile, upregulated expression of Runx2 and ALP genes was accompanied by increases in H3 acetylation levels in the promoter of the corresponding genes (Fig. 6A, B, D and E). We also examined DNA methylation in CpG islands in the promoter and exon 1 regions of these genes in parallel. Our results showed alterations in methylation levels in most of these genes in MSCs undergoing culture expansion, but they did not correlate to expressional changes of the corresponding genes except for ALP (Fig. 4C and D; Fig. 5B, C, E and F; Fig. 6 B, C, E and F).

### bFGF promotes MSC proliferation, suppresses MSC spontaneous differentiation and regulates histone acetylation

**Figure 7:**
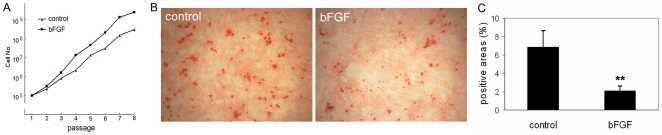
Effects of bFGF on MSC proliferation and spontaneous differentiation. (A) Growth curves of MSCs cultured in growth medium (control) and in growth medium supplemented with bFGF (bFGF). (B) MSCs cultured in growth medium (control) or in growth medium supplemented with bFGF (bFGF) for 3 days after Alizarin Red S staining. (C) Mean proportion of Alizarin Red S positive areas per field after quantification of 4 randomly selected fields (** *P*<0.01).

We proposed that alterations in MSC properties were largely caused by extrinsic signals and tested whether bFGF helped maintain the primitive features of MSCs. We found that MSCs grown in growth medium supplemented with bFGF had a 10 fold increase in cumulative cell number by passage 4 compared to MSCs grown in growth medium alone (Fig. 7A). When passage 6 MSCs grown in growth medium or in growth medium supplemented with bFGF were modestly induced in osteogenic induction medium for 3 days, the later had significantly less mineral deposition than the former (Fig. 7B and C), indicating that bFGF suppressed spontaneous differentiation of MSCs towards osteoblasts, but retained the potency of osteogenesis.

We then analyzed the influence of bFGF on epigenetic regulation in MSCs. We found that bFGF significantly suppressed alterations in histone H3 acetylation caused by *ex vivo* culture in TERT, Sox2, Oct4, Runx2 and ALP genes (Fig. 4 to [Fig pone-0020526-g005] 6), and this effect highly coincided with the effects of bFGF on MSC proliferation and spontaneous differentiation (Fig. 7). Meanwhile, we also examined DNA methylation in CpG islands in the promoter and exon 1 regions of these genes in parallel. Our results showed evident influences of bFGF on DNA methylation levels in Oct4, Runx2 and ALP, but not in TERT and Sox2 (Fig. 4 to [Fig pone-0020526-g005] 6). But these changes in DNA methylation did not correlate to expressional changes of the genes.

## Discussion

Due to encouraging effects of MSCs in tissue repair/regeneration demonstrated in animal studies, numerous clinical studies on MSC-based therapies for diverse diseases are underway. However, concerns over the consequences of altered properties of MSCs after *ex vivo* expansion remain. In this study, we found that MSCs underwent progressive spontaneous differentiation towards osteoblasts in MSCs undergoing regular culture expansion.

The molecular mechanisms underlying MSC spontaneous differentiation and aging are unclear. Previous studies have indicated that limited expansion of MSCs does not cause alterations in gene sequences [21], implying that alterations of MSCs *ex vivo* are probably not due to genetic instability. Therefore, in this study, we examined epigenetic changes in histone H3 acetylation and gene promoter DNA methylation. We found marked changes in histone H3 acetylation in K9 and K14 in TERT, Soc2, Oct4, Runx2 and ALP genes in late passage (P6) MSCs compared to early passage (P1) MSCs, and H3 acetylation levels coincided closely with gene expressional levels and MSC aging and spontaneous osteogenic differentiation, suggesting that histone H3 acetylation is an important mechanism in regulating MSC aging and differentiation. It has been known that posttranslational histone modifications participate in modulating the structure and function of chromatin and are critical in regulating gene transcription. Promoters of transcribed genes are enriched with hyperacetylation on the N-terminal tail of histone H3 [24]. Acetylation of K9 and K14 in histone H3 is required for the recruitment of TFIID [25], and TFIID binding to the promoter causes DNA bending and downstream translocation of the SWI/SNF-modified nucleosome, thus allowing the initiation of transcription [26]. It has been found that increases in levels of promoter histone H3 acetylation at lysine 9 in human hepatocellular liver carcinoma cells are accompanied with increases in overall gene expression levels [27]. In this study, we examined representative genes to reflect behavioral changes in MSCs during culture expansion. TERT enables cells to divide repeatedly and reduced expression of TERT is associated with cell aging [28]. Sox2 and Oct4 have long been known as key regulators of embryonic stem cell (ESC) self-renewal [29]. Runx2 is a key transcription factor associated with osteoblast differentiation, and the expression of Runx2 is a milestone for mesenchymal cells' commitment to osteoblasts [30], [31]. ALP is a characteristic gene expressed in osteoblasts [32].

Global histone acetylation levels have been examined in two recent studies. While no significant age related changes were detected in the global histone modification profiles of 4 histone core proteins (H2A, H2B, H3, and H4) in monkey BM derived MSCs in one study [33], genome-wide histone H3-K9 acetylation levels at gene promoters in human BM-derived MSCs were found to coincide well with overall mRNA expression levels in another [34]. These results suggest that MSC aging appears not to be associated with changes in global histone acetylation level but likely in gene promoter histone acetylation level. Indeed, in this study, we identified critical genes in which evident histone H3 modulations occurred in promoters in accordance with MSC aging, self-renewal and osteogenic differentiation. Our findings may facilitate the development of novel markers for early detection of MSC aging and spontaneous differentiation, thereby to ensure the quality of MSCs for clinical uses.

Recent studies suggest that DNA methylation is an important mechanism in regulating self-renewal and multipotency of HSCs and leukemia stem cells by silencing the expression of genes in differentiation [35]. In this study, we compared DNA methylation levels in CpG islands in promoter and exon 1 regions of genes involved in self-renewal including Sox2 and Oct4, proliferation and aging such as TERT and osteogenic differentiation including Runx2 and ALP in early versus late passage MSCs. To our surprise, though dysregulations in promoter DNA methylation exist in most of these genes, only changes in ALP gene coincided with gene expression levels. We obtained similar results in MSCs derived from cord tissues (data not shown). Some of our data are consistent with results published in a previous study, in which Runx2 was found hypermethylated in MSCs after long term culture [36]. Moreover, in the same study, a comparative methylation BeadChip microarray analysis in combination with cDNA microarray analysis did not reveal a clear correlation between the degrees of global CpG methylation and the levels of mRNA expression in long-term culture or aging MSCs [36]. Taken together, these results and ours suggest that dysregulations in promoter DNA methylation occur to aging MSCs, but may not coincide with gene expressional changes.

Previous studies suggest that aging and spontaneous differentiation of MSCs *ex vivo* are likely caused by inappropriate culture conditions [37], [38], [39], [40]. The physiological niche of MSCs is proposed to suppress spontaneous differentiation of MSCs into osteoblasts and facilitate their self-renewal. We proposed that potent extrinsic signals that alter MSC behavior will change the epigenetic status of corresponding genes. As a proof of concept, we examined the influence of bFGF, a potent cytokine that has been used as a supplement to MSC culture [41], on the histone acetylation and DNA methylation levels in MSCs. We found that supplementation of bFGF to the growth medium significantly promoted MSC proliferation and suppressed their spontaneous differentiation towards osteoblasts. Moreover, in accordance with these changes, bFGF treatment delayed the downregulation of TERT, Sox2 and Oct4, and suppressed the upregulation of Runx2 and ALP in culture MSCs. Along with expressional changes of these genes were corresponding alterations in histone H3-K9 and K14 acetylation levels, but not in promoter DNA methylation levels. bFGF has been known as an essential component of the regulatory niche in maintaining the pluripotency of ESCs, and inhibition of FGF signal pathway causes differentiation of ESCs [42], [43]. However, the mechanisms underlying the effect of bFGF in stem cell self-renewal are not fully understood. Our results show that bFGF regulates promoter histone H3 acetylation and consequently the expression of genes critical for stem cell self-renewal and osteogenic differentiation.

## Materials and Methods

### Cell isolation and culture

MSCs were isolated from human placenta as described previously [44], [45]. Briefly, term (38–40 weeks' gestation) placentas from healthy donors were harvested with written informed consent and the procedure was approved by the Ethics Committee of Xili Hospital. The placental tissue was washed several times with cold phosphate-buffered saline (PBS) and then mechanically minced and enzymatically digested with 0.25% trypsin-EDTA for 10 minutes at 37°C in a water bath. The digest was subsequently pelleted by centrifugation and resuspended in growth medium consisting of Dulbecco's modified Eagle's medium (DMEM, Gibco-Invitrogen) supplemented with 10% fetal bovine serum (FBS; Gibco-Invitrogen) and antibiotics. Cells were seeded on uncoated polystyrene dishes and incubated in growth medium at 37°C with 5% CO2. Medium was replaced every 2 days to reach 80% confluence. The cells that were lifted by incubating with 0.25% trypsin/EDTA for 2 min at 37°C were collected. In some cultures as indicated bFGF (R&D) at 5 ng/ml was added to the growth medium.

### Flow cytometry

MSCs were analyzed by flow cytometry analysis for immunophenotype [11]. Passage 3 cells were resuspended in PBS containing 1% bovine serum albumin (BSA) at 10^6^/ml. 100 µL cell aliquots were incubated with fluorescein isothiocyanate (FITC)- or phycoerythrin (PE)-conjugated monoclonal antibodies against CD73 (eBioscience), CD105 (endoglin), CD90, CD45, CD51, CD61 and CD34 (BioLegend), or control isotype IgG on ice for 30 minutes. 10,000 events were analyzed by flow cytometry (Becton Dickinson) using Cell Quest software.

### MSC differentiation assays

Passage 4 MSCs were incubated to differentiate into adipocytes, osteoblasts and chondrocytes in corresponding induction medium as previously described [1], [11]. After 3 weeks of culture with adipogenic induction medium containing 10^−6^ M dexamethasone, 10 µg/mL insulin, and 100 µg/mL 3-isobutyl-L-methylxantine (Sigma), cells were stained with Oil Red-O to detect lipid. Osteogenic medium contained 10^−7^ M dexamethasone, 50 µg/ml ascorbic acid, and 10 mM β-glycerophosphate (Sigma). Cultures at 3 weeks were stained using Alzarin Red for calcium deposition. For chondrocyte differentiation, MSC were cultured in DMEM (high glucose) containing 10^−7^ M dexamethasone, 50 µg/ml ascorbate-2-phosphate, 100 µg/ml pyruvate (Sigma), 10 ng/ml TGF-β1 (R&D Systems) and 50 mg/ml ITS+Premix (BD Biosciences, 6.25 µg/ml insulin, 6.25 µg/ml transferrin, 6.25 ng/ml selenious acid, 1.25 mg/ml bovine serum albumin, and 5.35 mg/ml linoleic acid). Cultures at 3 weeks were fixed and stained for alcian blue (Sigma).

### RNA extraction and Real-Time PCR

Total RNA was extracted from MSCs with TRIzol (Invitrogen) following the manufacturer's instructions. First-strand cDNA was prepared by reverse transcription with Superscript II reverse transcriptase (Invitrogen) and oligo(dT) primers and stored at −20°C. Real-Time PCR was performed using SYBR® Premix Ex Taq™ II (TaKaRa) on an ABI 7300 QPCR System. As an internal control, levels of glyceraldehyde-3- phosphate dehydrogenase (GAPDH) were quantified in parallel with target genes. Normalization and fold changes were calculated using the ΔΔCt method [11]. Primer sets are listed in Table 1.

**Table 1:** Primers for Real-Time PCR.

	FORWARD	REVERSE
Oct4	5′-GTATTCAGCCAAACGACCATC-3′	5′-CTGGTTCGCTTTCTCTTTCG-3′
TERT	5′-CGGAAGAGTGTCTGGAGCAA-3′	5′-GGATGAAGCGGAGTCTGGA-3′
Sox2	5′-GGGAAATGGGAGGGGTGCAAAAGAGG-3′	5′-TTGCGTGAGTGTGGATGGGATTGGTG-3′
Nanog	5′-AATACCTCAGCCTCCAGCAGATG -3′	5′-TGCGTCACACCATTGCTATTCTTC-3′
CD133	5′-ACATGAAAAG ACCTGGGGG-3′	5′-GATCTGGTGT CCCAGCATG-3′
Rex1	5′-GAAGAGGCCTTCACTCTAGTAGTG-3′	5′-TTTCTGGTGTCTTGTCTTTGCCCG-3′
Runx2	5′-ATGTGTGTTTGTTTCAGCAGCA-3′	5′-TCCCTAAAGTCACTCGGTATGTGTA-3′
Collagen-I	5′-CAGCCGCTTCACCTACAGC-3′	5′-TTTGTATTCAATCACTGTCTTGCC-3′
Osteocalcin	5′-GAAGCCCAGCGGTGCA-3′	5′-CACTACCTCGCTGCCCTCC-3′
Osteopontin	5′-CTCAGGCCAGTTGCAGCC-3′	5′-CAAAAGCAAATCACTGCAATTCTC-3′
ALP	5′-GACCCTTGACCCCCACAAT-3′	5′-GCTCGTACTGCATGTCCCCT-3′
BSP	5′-GCAGTAGTGACTCATCCGAAGAA-3′	5′-GCCTCAGAGTCTTCATCTTCATTC-3′
GAPDH	5′-GCACCGTCAAGGCTGAGAAC-3′	5′-TGGTGAAGACGCCAGTGGA-3′

ALP, alkaline phosphatase; BSP, bone sialoprotein.

### Chromatin immunoprecipitation

Chromatin immunoprecipitation (ChIP) assay was performed using an Acetyl-Histone H3 Immunoprecipitation Assay Kit (Millipore) following the manufacturer's protocol [46]. 1×10^6^ cells were used for each reaction. Histone acetylation was determined using specific antibodies against acetylated histone H3 at lysines 9 (K9) and 14 (K14), respectively (included in the kit). After chromatin immunoprecipitation, DNA was extracted with a standard procedure (phenol/chloroform/isoamilic alcohol 25∶24∶1), and subsequently measured by quantitative fluorescent PCR analysis using SYBR® Premix Ex Taq™ II (TaKaRa) on an ABI 7300 QPCR System. Primer targets were within 500 bp upstream of gene transcription start site and primer sets were as follows: TERT forward 5′-GGCTCCCAGTGGATTCGC-3′, reverse 5′-GGAGGCGGAGCTGGAAGG-3′; Sox2 forward 5′-AGTTGGACAGGGAGATGGC-3′, reverse 5′-AACCTTCCTTGCTTCCACG-3′; Oct4 forward 5′-CTTCCACAGACACCATTGCC-3′, reverse 5′-AGTCCCACCCACTAGCCTTG-3′; Runx2 forward 5′-GCTTCAATCCTTTCCTACAAAG-3′, reverse 5′-CGTGTGCAGTTTCCGACAG-3′; ALP forward 5′- TGTTGACAGACACAGAGACAGACG-3′, reverse 5′- GTCGGCATCTTCCTTCTGCG-3′. Data were analyzed using Percent Input Method (Invitrogen) followed the instruction.

### Genomic DNA extraction and bisulfite sequencing

MSCs were lysed in a lysis buffer containing 0.5% SDS, 0.1 M EDTA, 10 mM Tris–HCl, pH 8.0, and 100 ng/ml proteinase K, and incubated at 55°C overnight. Genomic DNA was purified by phenol/chloroform extraction. DNA (2 µg) from each sample was bisulfite converted using an EZ DNA Methylation-Gold™ Kit (Zymo research Corporation, Orange, CA, USA). To identify the methylation pattern of the genes, primers were designed to amplify the promoter and/or exon 1 region. The sequences of primers are as follows. TERT forward 5′- GGGATGTGATTAGATGTTCGGTT-3′, reverse 5′- ACCCAAAACTACCTCCAAAT-3′ (cover region −891∼−463); Sox2 forward 5′- GATTTTAATAAGAGAGTGGAAGGAA-3′, reverse 5′- CAAAACCAACCCTAACATTTT-3′ (cover region −421∼+21); Oct4 forward 5′-GTTAGAGGTTAAGGTTAGTGGGTGGGAT-3′, reverse AACACTAACCCCACTCCAACCTAAAAC-3′ (cover region −57∼+311); Runx2 forward 5′-GGGGTTAGAGTTTTTTTTTGTG-3′, reverse 5′- ACAAACACTAACCCCAAACC-3′ (cover region −384∼−101); ALP forward 5′-GGTTGAATTTTAATGGTAGGGT-3′, reverse 5′- CACCAAAAAAAACTCAATCA-3′ (+240∼+538). Touch down PCR was then carried out. The amplified products were gel-purified, and cloned into pMD-18T vectors (Takara). After that the recombinant plasmid was transformed into E. coli JM109. Seven to eleven clones for each gene were collected for sequencing [47].

### Statistical analysis

All values are expressed as mean ± SD. Student's paired *t* test was performed for comparison of data of paired samples and ANOVA was used for multiple group comparisons. A probability (*P*) value < 0.05 was considered significant.
